# Drought and exogenous abscisic acid alter hydrogen peroxide accumulation and differentially regulate the expression of two maize RD22-like genes

**DOI:** 10.1038/s41598-017-08976-x

**Published:** 2017-08-18

**Authors:** Kyle Phillips, Ndiko Ludidi

**Affiliations:** 1 Plant Biotechnology Research Group, Department of Biotechnology, Life Sciences Building, University of the Western Cape, Robert Sobukwe Road, Bellville, 7530 South Africa; 2 DST-NRF Centre of Excellence in Food Security, University of the Western Cape, Robert Sobukwe Road, Bellville, 7530 South Africa

## Abstract

Increased biosynthesis of abscisic acid (ABA) occurs in plants in response to water deficit, which is mediated by changes in the levels of reactive oxygen species such as H_2_O_2_. Water deficit and ABA induce expression of some RD22-like proteins. This study aimed to evaluate the effect of water deficit and exogenous ABA (50 µM ABA applied every 24 hours for a total of 72 hours) on H_2_O_2_ content in *Zea mays* (maize) and to characterise genes encoding two putative maize RD22-like proteins (designated ZmRD22A and ZmRD22B). The expression profiles of the two putative maize RD22-like genes in response to water deficit and treatment with ABA were examined in leaves. *In silico* analyses showed that the maize RD22-like proteins share domain organisation with previously characterized RD22-like proteins. Both water deficit and exogenous ABA resulted in increased H_2_O_2_ content in leaves but the increase was more pronounced in response to water deficit than to exogenous ABA. Lignin content was not affected by exogenous ABA, whereas it was decreased by water deficit. Expression of both RD22-like genes was up-regulated by drought but the ZmRD22A gene was not influenced by exogenous ABA, whereas ZmRD22B was highly responsive to exogenous ABA.

## Introduction

Abscisic acid (ABA) is a phytohormone involved in the induction of drought-responsive genes in *Arabidopsis thaliana* ^1^, *Oryza sativa* ^2^ and *Glycine max* ^3^. The induction of drought-responsive genes by ABA occurs through the binding of specific transcription factors, such as ABA-responsive element binding protein (AREB) and abscisic acid response element-binding factors (ABF), to *cis*-acting elements in the promoter regions of drought-responsive genes^4, 5^. However, there are several drought-induced genes which are not responsive to ABA. The induction of the expression of these genes involves the binding of specific transcription factors, such as dehydration responsive element binding (DREB) proteins, to *cis*-acting elements in promoters of these genes^6^.

Some of the drought-responsive genes which are responsive to ABA signals are the plant-specific RD22-like proteins, which are members of the BURP domain-containing protein family^7^. RD22-like proteins have been identified in *Arabidopsis thaliana* ^8^, *Brassica napus* ^9^ and G*lycine max* ^10^. The BURP domain-containing family of proteins is named after its primary sub-families BNM2, USP, RD22 and PG1β. These proteins have been identified in a variety of plant species where they are expressed under a diverse range of conditions and in various plant organs^7^. BURP domain-containing proteins are expressed during the early stage of microspore embryogenesis in *Brassica napus* L. (oilseed rape) seeds, as is seen with BNM2^11^. In *Vicia faba L*. (broad bean), USP (a non-storage seed protein with unknown function) is expressed during the early stages of both zygotic embryogenesis and *in vitro* embryogenesis^12^. BURP domain-containing proteins also regulate fruit ripening. PG1β, which is the non-catalytic β-subunit of polygalacturonase isozyme (PG), accumulates in ripening *Solanum lycopersium L*. (tomato), where they regulate pectin metabolism^13^. RD22 proteins are expressed in *Arabidopsis thaliana* in response to drought^14^. It is thus clear that BURP domain-containing proteins are involved in a range of metabolic and developmental processes, yet the exact mechanisms through which they function are only partially understood.

Specific structural characteristics shared by BURP domain-containing proteins include a C-terminal BURP domain made up of approximately 230 amino acids, with four conserved cysteine-histidine repeats ending in a tryptophan residue, characterized by a conserved X_5_-CH-X_10_-CH-X_23-27_-CH-X_8_-W (X is any amino acid) motif^15^. All BURP domain-containing proteins contain an N-terminal hydrophobic signal peptide which is followed by a conserved region used to distinguish between sub-families^16^. BURP domain-containing protein subfamilies are classified based on the number of short conserved segments in this region or the complete absence of the same region. BNM2-like subfamily proteins lack this region and have an N-terminal signal peptide linked to the BURP domain by a short conserved region immediately adjacent to the signal peptide^17^. USP-like and RD22-like subfamilies contain a variable region approximately 30 amino acids in length. The two subfamilies are distinguished from each other by the presence of 3 to 5 TxV repeat units in the variable region of RD22-like proteins whereas USP-like proteins have a variable region free of such repeat units^10, 15^. The presence of several repeats of a 14 amino acid sequence identifies the PG1β-like subfamily^17^.

This study reports the identification of two putative RD22-like proteins encoded by GRMZM2G446170 and GRMZM5G800586 in maize, designated ZmRD22A and ZmRD22B, respectively. Furthermore, the effect of drought and exogenous ABA on the expression of these genes is elucidated in maize leaves using semi-quantitative and quantitative PCR analysis. In light of the influence of drought and ABA on H_2_O_2_^18^, together with the link between H_2_O_2_ and cell wall lignification^19^, the study is extended to evaluate changes in H_2_O_2_ content and cell wall lignin content in maize leaves upon exposure to water deficit or treatment with ABA.

## Results

### ZmRD22A and ZmRD22B are BURP domain-containing proteins

**Figure 1:**
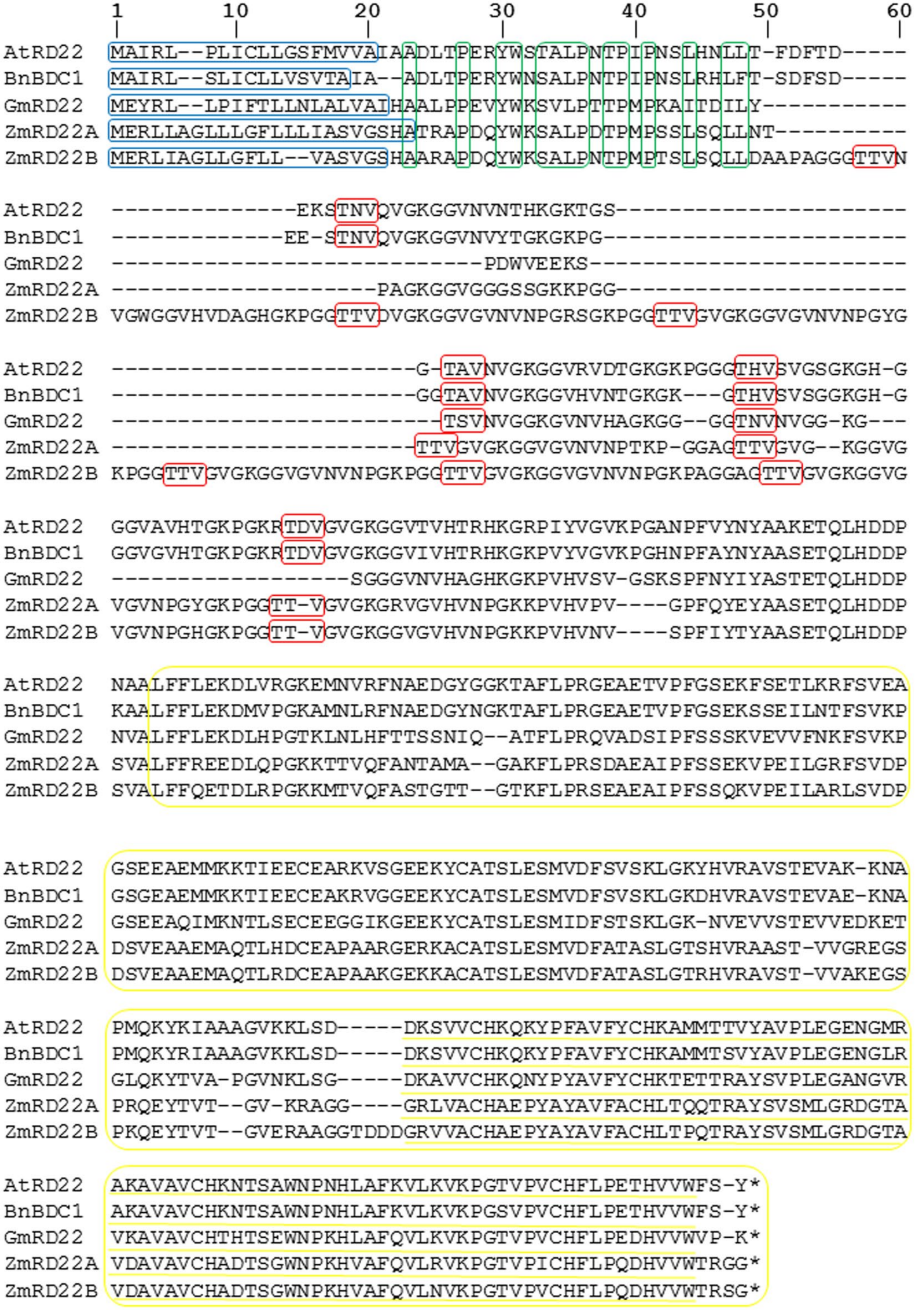
Sequence alignment and domain organization of five BURP domain-containing proteins. At5G25610 (AtRD22), AAQ57584 (BnBDC1), Glyma06g08540 (GmRD22), GRMZM2G446170 (ZmRD22A) and GRMZM2G800586 (ZmRD22B) were aligned, resulting in 136 identical sites (29% identity) and a pairwise identity of 52.4%. Similar domains and structural components were identified and are indicated as follows: the hydrophobic signal peptides are encircled in blue, the conserved amino acids in the conserved region are shown in green, The TxV (x is any amino acid) repeats are shown in red, the BURP-domains are shown in yellow and the conserved motif of the BURP-domain is underlined with yellow.

A signal peptide was identified in both the putative maize RD22-like proteins (namely ZmRD22A and ZmRD22B) as well as in all three of the reference BURP domain-containing proteins (namely *Arabidopsis thaliana* AtRD22, *Brassica napus* BnBDC1 and *Glycine max* GmRD22). In ZmRD22A, the signal peptide is located from amino acid 1 to 24 and gets cleaved between the 24^th^ and 25^th^ amino acid residues. Similarly ZmRD22B has a signal peptide ranging from amino acid 1 to 21 which is cleaved between the 21^st^ and 22^nd^ amino acid residues in the protein sequence, as indicated in Fig. 1 by the portion of sequence encased in blue. The hydrophobic signal peptide is followed by a region of approximately 25 amino acids (indicated in Fig. 1 in green) which is conserved in the putative maize RD22-like proteins as well as the three reference proteins. A variable region with 3 to 5 TxV repeats was identified, as shown in Fig. 1 (encased in red). Both ZmRD22A and ZmRD22B have 3 of these TxV repeats in their respective variable regions.

The most definitive characteristic identified was the BURP domain, which was present in the three reference RD22-like proteins as well as the putative maize RD22-like proteins. The BURP domains are indicated in Fig. 1 in yellow. In ZmRD22A, the domain spans from amino acid 160 to 374, and in ZmRD22B it spans from amino acid 236 to 455. The X_5_-CH-X_10_-CH-X_23-27_-CH-X_8_-W motif is present in all of the BURP domains identified and is underlined in yellow in Fig. 1. The alignment of protein sequences (Fig. 1) together with sequence similarity matrix analyses (Table 1), indicate that the maize proteins are 86% similar to each other and are more similar to soybean GmRD22 than the rest of the other BURP domain-containing proteins analysed here (Table 1). Nonetheless, all the proteins compared here have a similarity percentage of at least 50% (Table 1). Prediction of the subcellular localization of ZmRD22A and ZmRD22B proteins shows that they are both cell wall-localized, likely in association with the apoplast (Supplementary Fig. S1).

**Table 1:** Percentage identity matrix for the alignment of five BURP-domain containing protein sequences.

Target Sequence	Query Sequence				
	1	2	3	4	5
1 (AtRD22)	100%	87.34%	58.02%	50.42%	51.83%
2 (BnBDC1)	87.34%	100%	59.53%	50.42%	52.52%
3 (GmRD22)	58.02%	59.53%	100%	53.33%	53.71%
4 (ZmRD22A)	50.42%	50.42%	53.33%	100%	86.06%
5 (ZmRD22B)	51.83%	50.42%	53.71%	86.06%	100%

### ZmRD22A and ZmRD22B differ in the number of ABRE motifs

Analyses of the promoter region 2000 nucleotide bases upstream of the transcription start site of ZmRD22A and ZmRD22B revealed the presence of transcription factor binding sites which are involved in the induction of gene expression by ABA, namely occurrence of abscisic acid response element (ABRE) motifs (Table 2). However, only one ABRE was identified in the ZmRD22A promoter region and no coupling element was identified in the sequence. In the promoter region of ZmRD22B, several ABRE motifs that are sites for binding of AREB transcription factors, which mediate ABA responses, were identified.

**Table 2:** Identification of transcription factor binding sites involved in responses to abscisic acid.

Gene	TF Family	TF Name	Position	Strand	Similarity Score	Hit Sequence
ZmD22A	bZIP	AREB2	392	+	0.96	caaACGTGgg
ZmRD22B	bZIP	AREB2	230	+	0.95	gcCACGTgtcg
	bZIP	AREB2	233	−	1.00	gccACGTGtcg
	bZIP	AREB2	233	+	1.00	gccACGTGtcg
	bZIP	AREB2	506	−	0.75	ACAAGtat
	bZIP	AREB2	607	+	0.75	gcaCTTGT

### Water deficit and exogenous ABA affect H_2_O_2_ content

**Figure 2:**
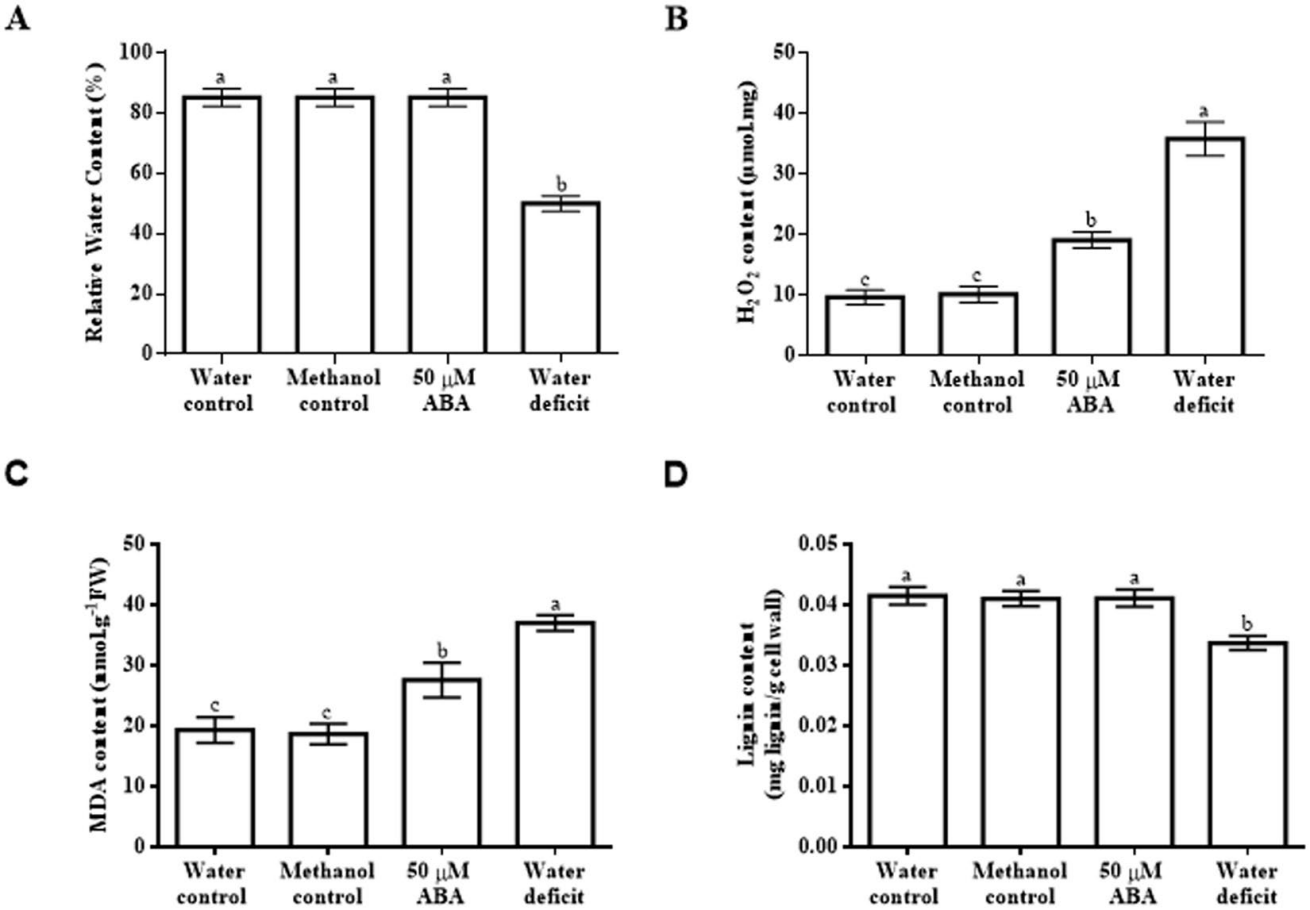
Effect of exogenous abscisic acid and water deficit on relative water content, H_2_O_2_ content, lipid peroxidation and lignin content in maize leaves. (**A**) Relative water content of maize leaves in response to ABA or water deficit. (**B**) Levels of H_2_O_2_ in maize leaves upon application of ABA or exposure to water deficit. (**C**) Changes in lipid peroxidation based on measuring MDA content in leaves. (**D**) Cell wall lignin content of maize leaves subjected to ABA treatment or water deficit. Data represent means of three independent experiments. The error bars signify standard errors, where bars with the same letters are statistically similar at *p* < 0.05.

Drought causes water deficit in plants, which can be evaluated by measuring the relative water content in leaf tissue. In this study, the plants grown under water deficit showed a 0.41-fold decrease in leaf relative water content (RWC) compared to leaf RWC of well-watered (water control) plants (Fig. 2A). Methanol and ABA did not alter the leaf RWC (Fig. 2A). Given the cross-talk between drought, ABA and H_2_O_2_, together with the influence of H_2_O_2_ on cell wall lignification, changes in H_2_O_2_ content, lipid peroxidation (as MDA content) and lignin content were measured. Addition of water and methanol had no effect on basal H_2_O_2_ content, whereas exogenous ABA caused a 0.98-fold increase in H_2_O_2_ content and water deficit resulted in a 2.6-fold increase in H_2_O_2_ content (Fig. 2B). Both the water control and methanol control had similar MDA contents but ABA treatment had a significant effect on MDA content, as indicated by a 0.49-fold increase in MDA content, whereas water deficit caused an even higher (0.98-fold) increase in MDA content (Fig. 2C). None of the treatments had an effect on cell wall lignin content except for water deficit, which caused a 0.13-fold decrease in lignin content (Fig. 2D).

### Drought and ABA influence the expression of ZmRD22A and ZmRD22B differently

**Figure 3:**
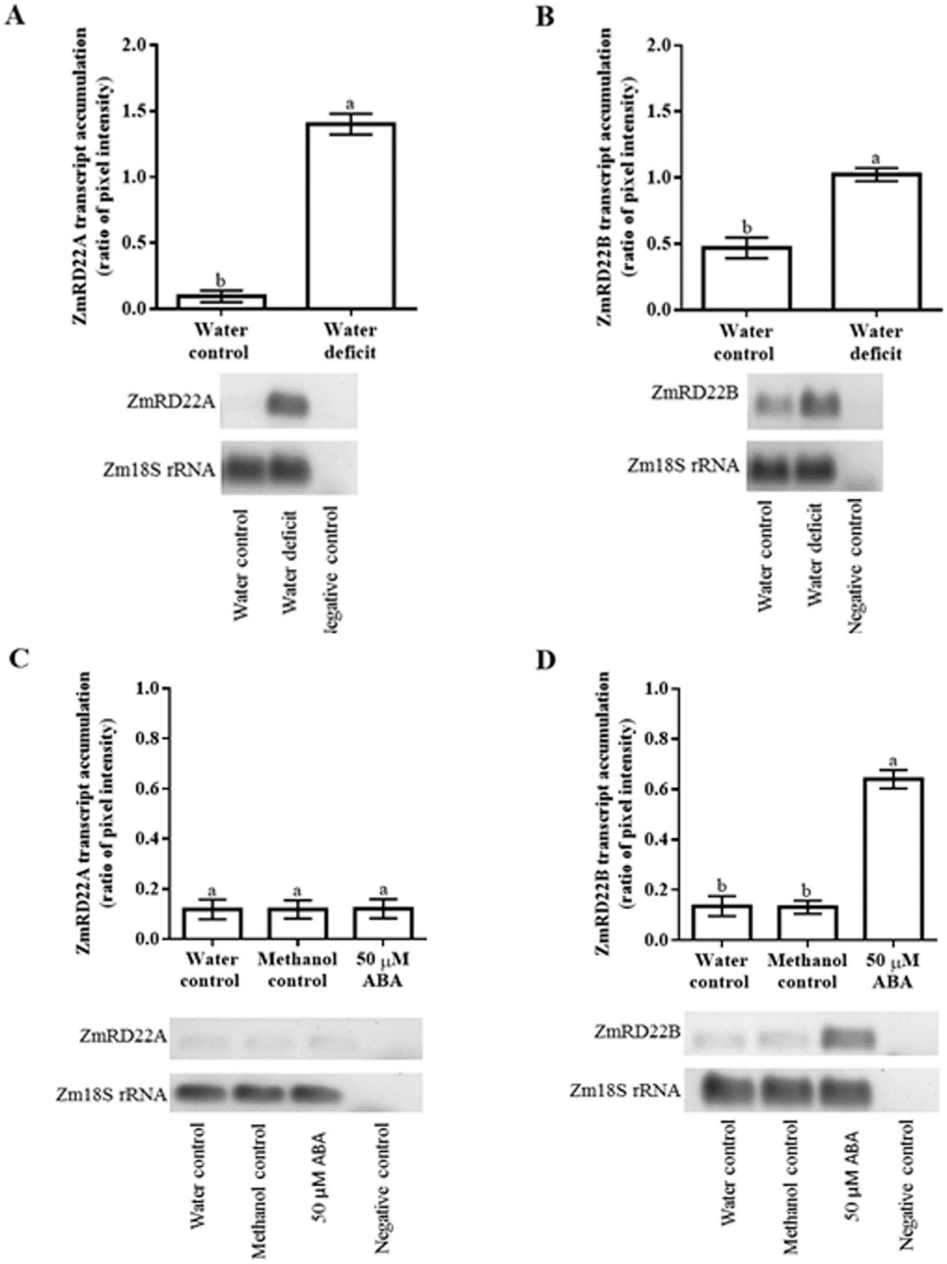
Semi-qRT-PCR analysis of the effect of water deficit or exogenous ABA on the expression of ZmRD22A and ZmRD22B in maize leaves. (**A**) Expression levels of ZmRD22A in response to water deficit. (**B**) Influence of water deficit on the expression of ZmRD22B. In both (**A** and **B**), controls are indicated by the first bar (water control). (**C**) Expression levels of ZmRD22A in response to exogenous ABA. (**D**) Response of ZmRD22B expression to exogenous ABA. In both (**C** and **D**), the controls are shown by the first and second bars (water and methanol controls, respectively) and the ABA treatment is shown by the third bar. In all cases, expression levels were measured relative to Zm18S rRNA expression. The error bars signify standard error in data from three independent experiments. Bars with the same letters are statistically similar where *p* < 0.05.

Semi-quantitative RT-PCR analysis was conducted to examine the effect of water deficit or exogenous ABA on the accumulation of ZmRD22A and ZmRD22B transcripts. Water deficit caused an 18-fold increase in the expression of ZmRD22A (Fig. 3A), whereas it resulted in a 1.6-fold increase in the expression of ZmRD22B (Fig. 3B). No change was detected in ZmRD22A transcript accumulation in response to the application of ABA (Fig. 3C). However, a 4.5-fold increase in ZmRD22B expression was detected in response to exogenous ABA (Fig. 3D).

**Figure 4:**
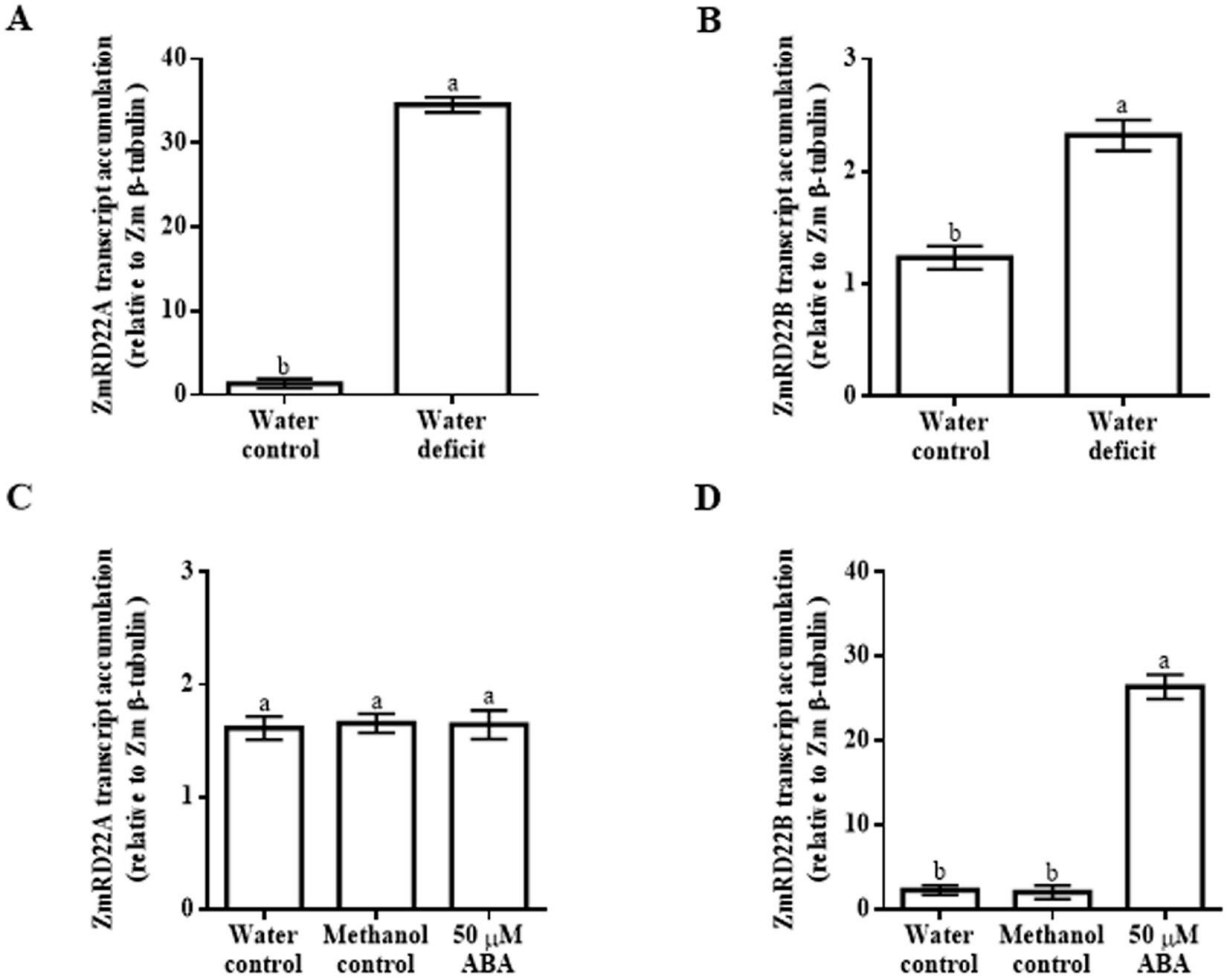
Quantitative RT-PCR analysis of ZmRD22A and ZmRD22B expression in response to exogenous ABA in maize leaves. (**A**) ZmRD22A transcript accumulation relative to maize β-tubulin in the 2^nd^ youngest leaves of maize seedlings treated with 50 µM ABA. (**B**) Representation of ZmRD22B transcript accumulation in the 2^nd^ youngest leaves, calculated in relation to Zmβ-tubulin, in response to exogenous treatment of maize seedlings with 50 µM ABA. In all the graphs, the water control is indicated by the first bar, the methanol control by the second bar and the ABA treatment by the third bar. Bars are means of three independent experiments and expression levels are signified as arbitrary values. The error bars signify standard deviation, bars with the same letters are statistically similar where *p* < 0.05.

To confirm the results obtained with semi-qRT-PCR, ZmRD22A and ZmRD22B transcript levels in response to water deficit and ABA treatment were measured using qRT-PCR. Three maize internal control genes, namely maize 18 S rRNA (Zm18S rRNA), maize β-tubulin (Zmβ-tubulin) and maize actin (ZmActin) were use as reference genes. The results obtained in relation to Zmβ-tubulin, which showed trends similar to those obtained with Zm18S rRNA and ZmActin, were used as a representative of the transcript accumulation of ZmRD22A and ZmRD22B. The expression of both ZmRD22A and ZmRD22B was up-regulated by water deficit, with ZmRD22A being more strongly induced (23-fold increase, Fig. 4A) than ZmRD22B (1.1-fold increase, Fig. 4B). The expression of ZmRD22A transcripts was not significantly affected by application of ABA (Fig. 4C). However, the expression of ZmRD22B increased 8.5-fold in response to ABA treatment (Fig. 4D).

### Expression of ZmRD22A and ZmRD22B varies in different regions of maize leaves

**Figure 5:**
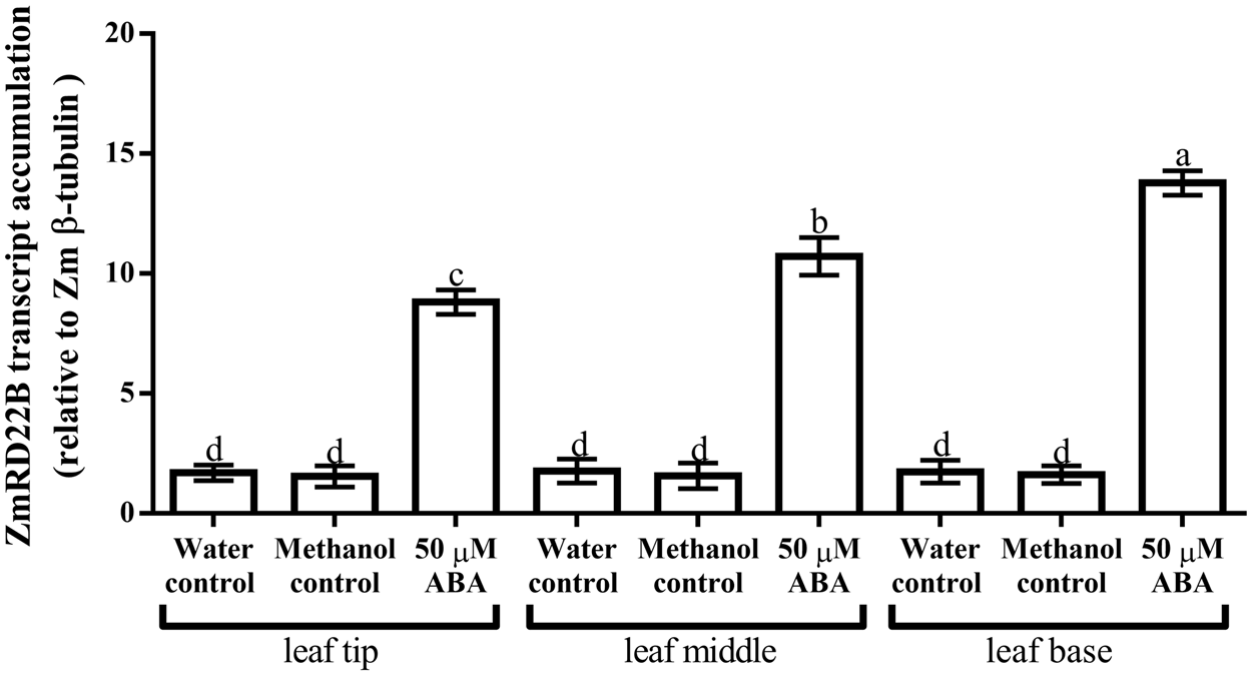
Expression of ZmRD22B in various regions of leaves in response to ABA treatments. Transcript accumulation of ZmRD22B, relative to Zmβ-tubulin, in various leaf regions of the 2^nd^ youngest leaves of ABA-treated maize seedlings were measured by qRT-PCR. Leaf regions examined were the tips of leaves, middle of the leaves and the base of the leaves. Error bars signify standard errors of means from three independent experiments, bars with the same letters are statistically similar, where *p* < 0.05.

**Figure 6:**
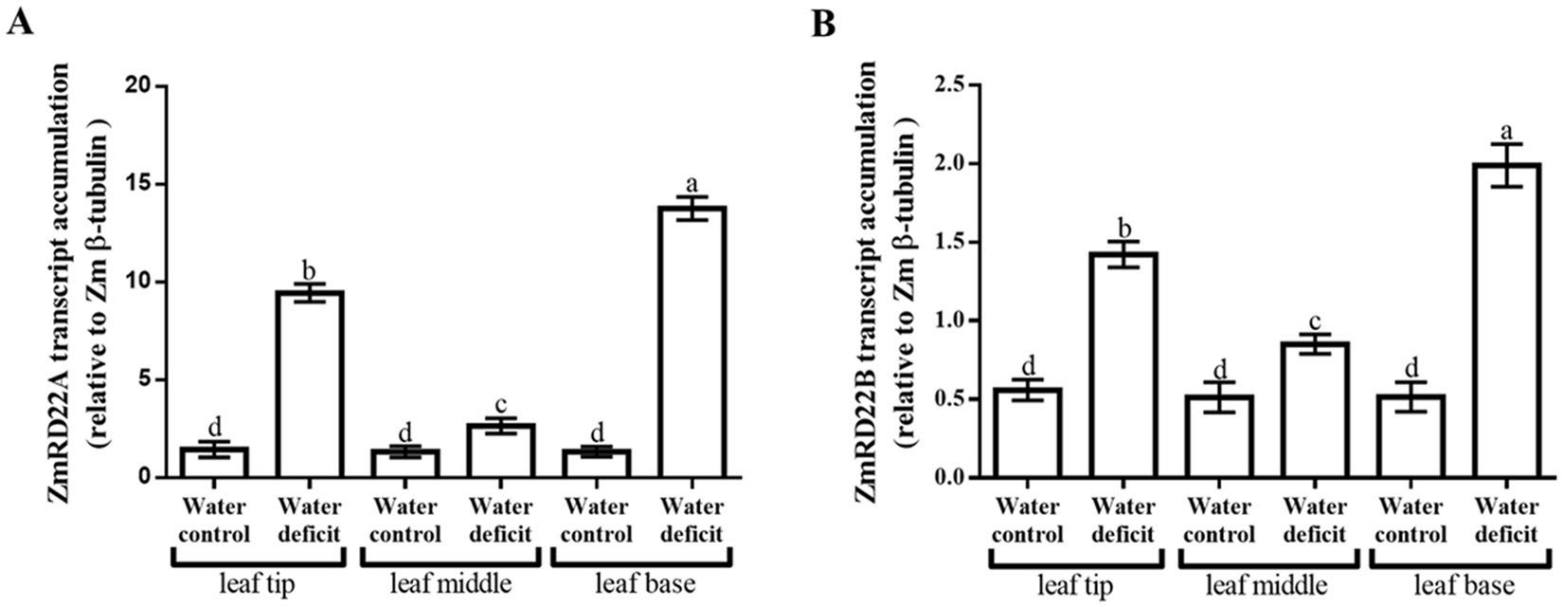
Expression of ZmRD22A and ZmRD22B in various regions of leaves in response to water deficit treatments. (**A**) Transcript accumulation of ZmRD22A in various leaf regions of the 2^nd^ youngest leaves of maize seedlings subjected to water deficit were measured by qRT-PCR. Leaf regions examined were the tips of leaves, middle of the leaves and the base of the leaves. (**B**) Expression of ZmRD22A in various leaf regions of the 2^nd^ youngest leaves of maize seedlings in response to water deficit. Expression was evaluated by qRT-PCR in the same leaf regions as in (**A**). Error bars signify standard errors of means from three independent experiments, bars with the same letters are statistically similar, where *p* < 0.05.

Upon observing that ZmRD22A expression was not influenced by treatment with ABA, the effect of ABA application on ZmRD22A transcript accumulation was not measured in the different regions of the leaf. On the other hand, analysis of the expression of ZmRD22B showed different levels of transcript accumulation for this gene in response to exogenous ABA when comparing the base, middle and tip of the leaves (Fig. 5A). In response to ABA treatment, a 3.2, 4.5 and 6.1-fold increase in ZmRD22B expression was observed at the tip, middle and base of the leaves, respectively (Fig. 6).

## Discussion and Conclusion

### Structural features of ZmRD22A and ZmRD22B suggests that they are BURP domain containing proteins

The bioinformatics analyses of two putative maize RD22-like proteins encoded by GRMZM2G446170 (ZmRD22A) and GRMZM5G800586 (ZmRD22B) was carried out in conjunction with three experimentally characterised RD22-like proteins, which were used as reference sequences identified in *Arabidopsis thaliana* (AtRD22)^14^ *, Brassica napus* (BnBDC1)^9^ and *Glycine max* (GmRD22)^16^. The analysis of potential signal peptides showed that ZmRD22A and ZmRD22B have highly similar signal peptide sequences, which may indicate a shared subcellular localization^20^. Predictive analyses on Plant-mPLoc show that both ZmRD22A and ZmRD22B are cell wall-localized proteins associated with the apoplast. This is in agreement with experimentally validated localization of RD22 proteins from soybean^16^ and tomato^17^, which have been shown to be localized in the cell wall. The TxV repeat units, which are specific to the variable region of the RD22 subfamily, were identified in all five sequences. The X_5_-CH-X_10_-CH-X_23-27_-CH-X_23-26_-CH-X_8_-W motif, found in all BURP domain-containing proteins, was also present in all five protein sequences. This confirms that both ZmRD22A and ZmRD22B belong to the BURP domain-containing protein family and that the two maize proteins belong to the RD22 subfamily.

### Differences in responses of ZmRD22A and ZmRD22B to exogenous ABA may be attributed to differences in the number of ABRE motifs in their promoters

Promoter analyses revealed the presence of transcription factor binding sites involved in induction of gene expression in response to ABA. The presence of several ABRE sites in the promoter of the ZmRD22B gene suggests possible regulation of this gene by ABA. Transcriptomic profiles of ZmRD22A and ZmRD22B show distinct differences in their responses to ABA, where ZmRD22A expression is not influenced by ABA and the expression of ZmRD22B is up-regulated by ABA. The occurrence of only one ABRE sequence in the promoter of ZmRD22A, together with the lack of a coupling element, justifies the inability of ABA to induce ZmRD22A because it has been demonstrated that induction of gene expression by ABA requires either several ABRE motifs or a single ABRE motif occurring simultaneously with a coupling element^21–23^. Similarly to some RD22-like genes, the expression of ZmRD22B was up-regulated by ABA. This up-regulation is seen, for example, in some soybean and Arabidopsis RD22-like genes^16, 24^. The differences in accumulation of ZmRD22B transcripts in different regions of the leaves suggests differential regulation of ABA responses in these regions, likely due to differences in the expression of transcription factors regulating ABA responses in these regions. The differences in *cis*-acting elements occurring in the putative promoters of ZmRD22A versus ZmRD22B supports the observed differences in the responses of these two genes to exogenous ABA, with the ZmRD22A promoter having only one ABRE but no coupling element (and thus is not up-regulated by ABA) whereas the ZmRD22B promoter has multiple ABRE motifs (and thus is responsive to ABA). This is in agreement with previous works where it was shown that occurrence of a single ABRE in a promoter of a gene is insufficient for induction of the expression of that gene by ABA unless there is a coupling element (CE) in proximity to the ABRE^21–23^. Alternatively, multiple ABRE motifs, in the absence of a CE, are required for induction of gene expression by ABA^21–23^, as observed for ZmRD22B. This justifies the ABA-independent regulation of the expression of ZmRD22A and the ABA-regulated expression of RD22B.

On the other hand, despite the contrasting responses of these two maize RD22-like genes to ABA, both are up-regulated by drought. Drought-induced expression of genes in an ABA-independent manner has been demonstrated for several genes^6^, justifying that the lack of ABRE and CE motifs in ZmRD22A does not exclude this maize RD22-like gene from responding to water deficit stress. It is possible that the stronger response of ZmRD22A to water deficit, compared to the response of ZmRD22B to water deficit, likely involves differences in binding affinities of transcription factors that regulate drought-induced expression or different levels of expression of such transcription factors in response to water deficit. Such transcription factors could include, among others and in addition to AREB proteins, the DREB family of proteins. The difference in transcription factor binding affinities could, for example, be due to variations in the number and nature of water deficit-related *cis*-acting elements in the two genes. This hypothesis could also explain the differences in the level of drought-induced expression of the two genes in the various regions of the leaves. Further study of the regulation of the two genes by drought is required to understand the molecular machinery that will lend credence to this hypothesis and will involve detailed *in silico* and *in vivo* functional analyses and classification of transcription factor-encoding genes co-expressed/co-localized with the two RD22-like genes in response to drought, followed by corresponding protein-DNA interaction studies.

### H_2_O_2_ levels induced by water deficit and exogenous ABA are sufficient to cause lipid peroxidation

There is evidence for cross-talk between ABA signals and H_2_O_2_ production in plant tissue^18, 25^, together with evidence for a role of H_2_O_2_ in causing lipid peroxidation^26^ and enhancing cell wall lignification^27^. It was on this basis that H_2_O_2_ content, MDA content and lignin content were measured. The findings made in this study show that maize seedlings supplied with water or methanol had similar leaf H_2_O_2_ and MDA contents, whereas water deficit and exogenous ABA resulted in increased H_2_O_2_ and MDA contents. This is in agreement with previous results showing the induction of H_2_O_2_ production in response to ABA and drought in a study examining the involvement of ABA-induced H_2_O_2_ biosynthesis in stomatal guard cell aperture size in response to drought stress^25^. Indeed, it is well established that exogenous ABA induces accumulation of H_2_O_2_ and that this is mediated via the induction of the expression of genes encoding NADPH oxidase in Arabidopsis and cotton in response to ABA, in both the absence and presence of abiotic stress^28–30^.

The fact that the increase in H_2_O_2_ content caused by water deficit is more pronounced than the increase caused by exogenous ABA, with corresponding increases in lipid peroxidation, suggests that the degree of water deficit in this study triggers a signalling response that may potentiate ROS generation to a higher extent than ABA alone. Given the role of ABA in influencing H_2_O_2_ content and the influence of H_2_O_2_ in cell wall lignification, cell wall lignin content was measured in maize seedlings subjected to water deficit or treated with ABA. In this study, application of ABA did not affect cell wall lignin content whereas water deficit reduced lignin content. This suggests that the level of induction of H_2_O_2_ production by the ABA treatment was not sufficient to cause changes in cell wall lignin content whereas water deficit induced H_2_O_2_ production to a level high enough to impact on lignin content. There is evidence showing that drought may either increase lignin content in some maize lines or decrease it in other maize lines^31^. Further investigation on the regulation of lignin content in response to drought in maize is thus required in order to clarify the effect of drought on lignin content and the molecular mechanisms involved in determining such effect. Besides H_2_O_2_, water deficit may act in concert with other signalling events to trigger the observed decrease in in lignin content.

We conclude that both ZmRD22A and ZmRD22B are drought-inducible RD22-like proteins and that only the expression of ZmRD22B of the two is ABA-inducible as a consequence of occurrence of the required *cis*-acting elements in the promoter of ZmRD22B. Given that the bioinformatics analysis suggests that both ZmRD22A and ZmRD22B are similar to RD22 proteins involved in drought responses, together with the involvement of ABA in drought responses, further characterisation of ZmRD22A and ZmRD22B will involve examining the physiological function of these genes in relation to maize responses to drought. Such study is of high relevance to improvement of maize tolerance to drought.

## Methods

### Determination of transit peptide cleavage sites and sub-cellular localization

The cleavage site for signal peptide removal was determined in five protein sequences using the SignalP4.1 server (http://www.cbs.dtu.dk/services/SignalP/). The five protein sequences used were ZmRD22A and ZmRD22B (the genes of interest) together with Glyma6G081100 (GmRD22), AT5G25610 (AtRD22) and AY293830 (BnBDC1) which were used as reference proteins. Identification of signal peptide cleavage sites was carried out using eukaryotic organism grouping and default D-cut-off values which are optimised for correlation^32^. Subcellular localization was predicted using the full length protein sequence as a query on Plant-mPLoc (http://www.csbio.sjtu.edu.cn/bioinf/plant-multi/).

### Identification of BURP domains

The presence of BURP domains in ZmRD22A and ZmRD22B was detected using MyHits SIB motif scan (http://myhits.isb-sib.ch/cgi-bin/motif_scan). The protein sequences for GmRD22, AtRD22 and BnBDC1 were used in the same program to determine the accuracy of the domain identification provided by motif scan. The protein sequences were subjected to Pfam hidden Markov models (HMMs) (http://pfam.xfam.org/) to identify all known motifs present in these protein sequences.

### Sequence alignment of BURP domain-containing RD22 proteins

Protein sequences of ZmRD22A, ZmRD22B and the three reference proteins (namely AtRD22, GmRD22 and BnBDC1) were obtained from Phytozome V10.3 and Genbank (http://phytozome.jgi.doe.gov/pz/portal.html and http://www.ncbi.nlm.nih.gov/genbank/, respectively). The protein sequences were imported into Geneious V8.1.7. (www.geneious.com) on which a global pairwise alignment using Blosum62 scoring matrix was carried out. The sequence alignment was then used to highlight structural components and domain organisation across the five gene sequences.

### Promoter analyses

A 2000 bp region upstream of the transcription start site of ZmRD22A and ZmRD22B genomic sequences was obtained from Phytozome V10.3 (http://phytozome.jgi.doe.gov/pz/portal). These sequences were then analysed on the PlantPan 2.0 plant promoter analysis tool (http://plantpan2.itps.ncku.edu.tw/promoter.php). Using the PlantPan transcription factor library specific for maize, possible transcription factor binding sites were identified.

### Plant growth

Maize (*Zea mays* L. cv CAP9001) seeds (donated by Capstone Seeds Pty Ltd, Howick, South Africa) were surface-sterilized in 0.35% sodium hypochlorite for 10 min and then rinsed four times with sterile distilled water. The seeds were imbibed in sterile distilled water for 1 hour and germinated on sterile moist filter paper in the dark at 23 °C. Germinated seeds with radicles that were approximately 3–5 mm were sown in 3 L of moist (water potential of −0.03 ± 0.003 MPa as determined using the WP4C Dew Point PotentiaMeter hygrometer from Degacon Devices Inc., Pullman, WA, USA) Promix Organic (Windell Hydroponics, Durbanville, South Africa) in 20 cm diameter plastic pots. The Promix Organic was supplemented with 10 mM 2-(N-Morpholino) ethansulfonic acid (MES), pH 6.0, to maintain the desired pH. For the water deficit experiments to mimic drought, the amount of water added to the Promix Organic monitored to give moisture levels that resulted in Promix Organic with a water potential of −0.35 ± 0.03 MPa before sowing the germinated seeds. For the controls and the ABA treatments, the Promix Organic was kept moist by watering with 100 ml of distilled water on the pot tray on which the pot is placed, at two days intervals until plants (one plant per pot) reached the V2 stage of vegetative growth (when there were two leaves with a well-defined/clearly visible collar on each leaf). For the water deficit, no further water was added to the Promix Organic (Ψ = −0.35 ± 0.03 MPa) after sowing the germinated seeds. Growth conditions in the growth room were maintained as 26/19 °C day/night temperature cycle under a 16/8 h light/dark cycle at a photosynthetic photon flux density of 400 μmol photons.m^−2^.s^−1^ during the day phase.

### Treatment of Plants and Measurement of Relative Water Content

Plants subjected to water deficit (one plant per pot, 16 pots per experiment) were not treated in any way other than as described above. These water deficit-treated plants were left in the Promix Organic until they entered into early V3 stage (two leaves with a well-defined/clearly visible collar on each leaf and one more leaf starting to show a poorly defined collar), which was 20 days after sowing of germinated seeds. At this stage of growth of the water deficit-treated plants, the seedlings were carefully removed from the Promix Organic, then the 2^nd^ youngest leaves were separated from the rest of the seedlings and several of these leaves were divided at the base (5 cm region at the base of the leaf), the middle (5 cm section from the middle of the leaf) and the tip (5 cm region from the tip of the leaf). Some of the 2^nd^ youngest leaves were left intact after separation from the rest of the seedlings. The intact leaves and leaf regions were snap-frozen in liquid nitrogen and stored at −80 °C for use in all experiments. Water potential of the Promix Organic in the root zone at this stage of harvesting was measured using the WP4C Dew Point PotentiaMeter hygrometer and determined to be −0.85 ± 0.09 MPa (average from 8 pots sampled randomly). Once the rest of the other seedlings (except for the water deficit treatment) completed the V2 stage, they (one plant per pot, 16 pots per experiment) were treated with 100 ml of 50 µM abscisic acid (ABA) in 10 mM MES, pH 6.0. Two separate subsets at the same stage were supplied with 10 mM MES, pH 6.0 (one plant per pot, 16 pots per experiment) or 0.05% methanol in 10 mM MES at pH 6.0 (one plant per pot, 16 pots per experiment) to act as a controls since the ABA was prepared in methanol and treatments with ABA in this set of experiments result in a final methanol concentration of 0.05%. The seedlings were treated in this way every 24 hours for a total of 72 hours, at which time they were in early V3 stage (which was 14 days after sowing of germinate seeds in the case of all controls and the ABA-treated plants).

The single concentration of ABA was chosen based on previous work where it was shown that low ABA concentrations (frequently 50 or 100 µM exogenous ABA) caused changes in expression of various ABA-responsive genes^16, 29, 30, 33, 34^ whereas higher concentrations were avoided because of the involvement of ABA in modulation of plant senescence^35^. The single time point for the treatment was selected on the basis of previous reports showing that RD22 transcript profiles in other plant species responded significantly to exogenous ABA only at 24 hours of application and up to 144 hours from treatment^16, 29^. After 72 hours of ABA or methanol treatment, the leaves of seedlings at early V3 stage were removed from the Promix Organic and processed as described above for leaves of seedlings that were subjected to water deficit. A 2 cm long region was cut, between the mid-vein and the edge, from the middle of some (four samples from different plants of each treatment) of the 2^nd^ youngest leaves from the water controls, methanol controls, ABA-treated and water deficit-subjected plants were used to measure leaf relative water content (RWC). For the RWC measurement, the 2 cm long regions were weighed to determine their fresh weights. The cuttings were subsequently placed in Petri dishes containing distilled H_2_O and incubated under ambient light at room temperature for 4 hours. The surface water on the leaves was removed by briefly patting the leaf sample in-between filter paper. The leaf samples were then weighed to determine turgid weight. Subsequently, the leaves were dried at 80 °C for 48 hours. The dried leaves were immediately placed in a desiccator and weighed to determine their dry weights. RWC was calculated according to RWC (%) = [(W − DW)/(TW − DW)] × 100, where W is fresh weight, TW is turgid weight and DW is dry weight.

### Determination of hydrogen peroxide content

Water deficit and ABA is known to cause changes in reactive oxygen species generation in plants^28, 30, 36^. In order to determine if the water deficit or ABA treatment affects H_2_O_2_ accumulation in the maize seedlings, the H_2_O_2_ content in the 2^nd^ youngest leaves of the seedlings was measured. For this measurement, leaves were ground into fine powder in liquid nitrogen and the H_2_O_2_ content was measured using a previously described method based on reaction with iodide ions^36^. In this method, 100 mg of leaf material was homogenized in 400 µl of cold 6% (w/v) trichloroacetic acid (TCA). The homogenate was then subjected to centrifugation at 12,000 × *g* for 30 min at 4 °C. The supernatant was taken as TCA extract and 50 µl of the TCA extract was mixed with 150 µl of reaction buffer containing 5 mM K_2_HPO_4_ at pH 5.0 and 0.5 M KI. Reactions were incubated at 25 °C for 20 min. Absorbance readings were recorded in triplicate at 390 nm. H_2_O_2_ content was calculated using a standard curve constructed with the absorbance of H_2_O_2_ standards read at an absorbance of 390 nm.

### Measurement of lipid peroxidation

In consideration of the fact that changes in H_2_O_2_ content could result in lipid peroxidation, the degree of lipid peroxidation was estimated by measuring malondialdehyde (MDA) content as previously described^37, 38^. Leaf tissue was ground into a fine powder in liquid nitrogen, 100 mg of which was homogenised in 400 µl of cold 6% (w/v) TCA. The leaf material was pelleted by centrifugation at 12, 000 × *g* for 30 min at 4 °C. The assay was performed by coalescing 100 µl of the TCA extract with 400 µl of 0.5% (w/v) 2-thiobarbituric acid (TBA) prepared in 20% TCA. The homogenate was incubated for 30 min at 95 °C. The reaction was stopped by cooling on ice for 10 min. The thiobarbituric acid reactive substances (TBRAS), reflective of MDA, were detected by reading their absorbance at 532 nm and subtracting nonspecific absorbance at 600 nm. The amount of MDA was calculated using a molar extinction coefficient of 155 mM^−1^cm^−1^ and expressed as nmol.g^−1^ of fresh weight.

### Lignin content assay

Given that H_2_O_2_ participates in cell wall lignification and this is linked to ABA-induced H_2_O_2_ generation^22^, the lignin content in the 2^nd^ youngest leaves of water deficit-treated, ABA-treated and control *Z*. *mays* seedlings was measured using the acetyl bromide method^39^. Protein-free cell wall preparations were obtained by grinding leaf material into a fine powder in liquid nitrogen. The ground leaf material (300 mg) was homogenised with 3 ml of 50 mM potassium phosphate buffer, pH 7.0. The Homogenate was centrifuged at 1 400 × *g* for 5 min to pellet the leaf material. The pellet was washed twice with 50 mM potassium phosphate buffer at pH 7.0. The pellet was then washed three times in 50 mM potassium phosphate buffer (pH 7.0) containing 1% (v/v) Triton X-100. The pellet was washed twice in 1 M NaCl made up with 50 mM potassium phosphate buffer (pH 7.0). The material was pelleted by centrifugation at 1400 × *g* for 5 min between each wash step. The pellet was washed twice with acetone, after which it was dried at 60 °C for 24 hours. This protein-free cell wall isolate (20 mg) was homogenized in 500 µl of 25% (v/v) acetyl bromide (made up in glacial acetic acid). The homogenate was incubated for 30 min at 70 °C and then cooled on ice for 5 min. To the homogenate, 900 µl of 2 M NaOH, 100 µl of 5 M hydroxylamine-HCl and 6 ml glacial acetic acid were added. The solution was centrifuged at 1400 × *g* for 5 min and the absorbance was measured at 280 nm. A blank was prepared containing all of the assay components excluding the protein-free cell wall isolate. Lignin content was calculated using a standard curve of alkali lignin (Sigma-Aldrich) and the results were expressed as mg lignin.g^−1^ cell wall.

### Total RNA isolation and first stand cDNA synthesis

Total RNA was extracted from the 2^nd^ youngest leaves of *Z*. *mays* seedlings exposed to water deficit or treated with 50 µM ABA or from the control treatments. Leaf material was ground to a fine powder in liquid nitrogen. Total RNA was isolated from 50 mg of ground plant material using the Direct-Zol™ RNA miniprep kit (Zymo Research) according to the instructions of the manufacturer. RNase-free DNase I (Zymo Research) was used to remove DNA from the isolated RNA as specified by the manufacturer. RiboLock^®^ RNase Inhibitor (Thermo Scientific) was added to prevent RNase-mediated degradation of the RNA. First strand cDNA synthesis was carried out using 500 ng of total RNA and the RevertAid™ Reverse Transcriptase kit (Thermo Scientific) as specified by the manufacturer.

### Semi-quantitative PCR analysis of ZmRD22A and ZmRD22B transcript levels

PCR amplification of ZmRD22A and ZmRD22B was carried out independently using 2 µl of first strand cDNA synthesised as previously described. The genes were amplified using TrueStart^™^ Taq (Thermo Scientific) as described by the manufacturer. The internal control gene maize 18 S rRNA (Zm18S rRNA) was PCR-amplified in the same way. Sequences of the gene-specific primers used in the PCR reactions are provided in Supplementary Table S1. To ensure that the reactions occurred in the PCR exponential phase, the reaction was conducted for 15, 20, 25 and 30 cycles, thus allowing for the earliest cycle number to be identified at which differences in transcript numbers are detectable. The PCR products were size-fractionated on a 1% agarose gel and visualised using 1X GelRed^™^ (Biotium Inc.). Individual gels from three independent experiments were used for expression analysis on the basis of densitometry. Densitometric analyses were done using the Spot Denso tool of AlphaEase FC Imaging Software (Alpha Innotech Corporation). Transcript accumulation was expressed as ratios relative to the values of the control samples, with densitometry-derived values of Zm18S rRNA as the reference.

### Quantitative real-time PCR analysis of ZmRD22A and ZmRD22B transcript accumulation

Quantitative measurement of ZmRD22A and ZmRD22B transcript level changes in response to water deficit or ABA treatment was carried out independently using quantitative PCR (qPCR). The qPCRs for water deficit, ABA treatments, methanol controls and water controls were done in triplicate using Luminaris Color HiGreen^™^ Low ROX qPCR master mix (Thermo Scientific) on the cDNA isolated as described above, as described by the manufacturer. Similar qPCR reactions were set up for three internal control genes, namely Zm18S rRNA, maize actin (ZmActin) and maize β-tubulin (Zmβ-tubulin). The sequences of the primers for these internal control genes are provided in Supplementary Table 1). Transcript accumulation levels were expressed as ratios relative to the values of the control samples, with the transcript accumulation levels of the internal control genes as the reference. The transcript levels for ZmRD22A and ZmRD22B were also measured in a similar manner in various leaf regions (tip, middle and base) from the 2^nd^ youngest leaves of plants which were subjected to water deficit or treated with ABA, methanol or water.

### Statistical analysis

The statistical validity of all the data was tested by means of a One-way analysis of variance (ANOVA) and the Tukey-Kramer test at 5% level of significance was completed to compare the means using GraphPad Prism 6.01 software (GraphPad Software Inc.).

## Electronic supplementary material

Supplementary Information 
