# Supplementary material for: Drought and exogenous abscisic acid alter hydrogen peroxide accumulation and differentially regulate the expression of two maize RD22-like genes
Source: Sci Rep. 2017 Aug 18;7:8821. doi: 10.1038/s41598-017-08976-x (PMC5562893; doi:10.1038/s41598-017-08976-x)
Supplement: Supplementary file 1 — Supplementary Information [file 41598_2017_8976_MOESM1_ESM.pdf]

Title: Drought and exogenous abscisic acid alter hydrogen peroxide accumulation and differentially regulate the expression of two maize RD22-like genes

Authors: Kyle Phillips[1] & Ndiko Ludidi[1,2]*

Affiliations:
[1]Plant Biotechnology Research Group
Department of Biotechnology
Life Sciences Building
University of the Western Cape
Robert Sobukwe Road
Bellville, 7530
South Africa

[2]DST-NRF Centre of Excellence in Food Security
University of the Western Cape
Robert Sobukwe Road
Bellville, 7530
South Africa

***Corresponding Author:** nludidi@uwc.ac.za

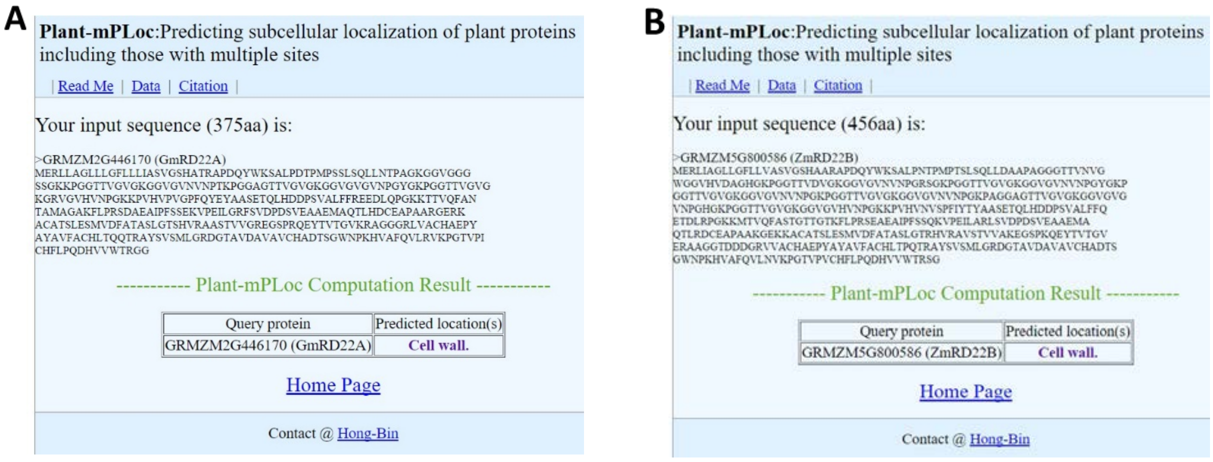

Figure S1: Prediction of the subcellular localization of maize RD22 proteins. (a) Subcellular localization of ZmRD22A based on Plant-mPLoc prediction. **(b)** Subcellular localization of ZmRD22B based on Plant-mPLoc prediction.

Supplementary Table S1: Primer sequences used in semi-quantitative and quantitative PCR analysis.

Gene name	Forward primer	Reverese primer
ZmRD22A	5'-GCG GGC GGG CGG CGG GCG CCT G-3'	5'-TCA GCCGCC GCG GGT CCAGAC GAC G-3'
ZmRD22B	5'- CGA CGA CGA CGG CCG GGT CGT G -3'	5'- TCA GCC GCT GCG GGT CCA GAC GAC GTG -3'
Zm18s rRNA	5'-CCA TCC CTC CGT AGT TAG CTT CT -3'	5'-CCT GTC GGC CAA GGC TAT ATA C-3'
Zm β-tubulin	5'- AGC CCG ATG GCA CCA TGC CCA GTG ATA CCT -3'	5'- AAC ACC AAG AAT CCC TGC AGC CCA GTG C -3'
Zm Actin	5'- GTG ACA ATG GCA CTG GAA TG -3'	5'- GAC CTG ACC ATC AGG CAT CT -3'
